# 扫描及重建条件对肺结节三维容积及CT值定量影响的体模研究

**DOI:** 10.3779/j.issn.1009-3419.2017.08.10

**Published:** 2017-08-20

**Authors:** 大同 苏, 磊 冯, 英健 姜, 颖 王

**Affiliations:** 1 300052 天津，天津医科大学总医院放射科 Department of Radiology, Tianjin Medical University General Hospital, Tianjin 300052, China; 2 300000 天津，天津市西青医院放射科 Department of Radiology, Tianjin Xiqing Hospital, Tianjin 300000, China

**Keywords:** 肺结节, 体模, 容积分析, CT值, 计算机X线体层摄影, Pulmonary nodules, Phantom, Volumetric measurement, CT value, Computed tomography

## Abstract

**背景与目的:**

计算机断层扫描（computed tomography, CT）随访评估结节体积及密度变化是临床针对不定性肺结节采用的常用策略。在保证测量精度前提下降低CT剂量是需要考虑的问题。本研究旨在评估不同管电流及重建算法对肺体模结节容积定量及CT值测量的影响。

**方法:**

应用64排螺旋CT，管电压120kV，7种管电流（10 mA、20 mA、50 mA、80 mA、100 mA、150 mA、350 mA）对肺结节体模进行扫描，采用滤波反投影（filtered back projection, FBP）、自适应迭代重建（ASIR: 30%, 50%, 80%）算法进行重建，获取28套CT图像。应用肺结节分析软件对3种直径（2.5 mm, 5 mm, 10 mm）、三种CT值（-100 HU, 60 HU, 100 HU）共9个球型结节测量容积及平均CT值数据。应用重复测量方差分析评估不同管电流及原始数据重建算法对容积及CT值测量的影响。

**结果:**

直径为2.5 mm结节的容积测量相对误差（100.8%±28%）及三维CT值绝对误差（-756±80）HU最大；直径为5 mm及10 mm结节的容积相对误差小[(-0.9%±1.1%)vs(0.9%±1.4%)]，但CT值绝对误差大[(-243±26)HU vs(-129±7)HU]。针对直径为5 mm及10 mm结节使用重复测量方差分析结果显示，应用不同管电流及原始数据重建算法时容积测量相对误差没有显著性差异（*F*=5.60, *P*=0.10 *vs* *F*=11.13, *P*=0.08），三维CT值的绝对误差有显著影响（*F*=34.79, *P* < 0.001 *vs* *F*=156.14, *P* < 0.001）。

**结论:**

不同管电流及重建算法对直径5mm及10 mm的结节容积定量影响很小，因此较低管电流及迭代重建算法可以应用在5 mm以上肺结节的CT随访中。结节分析软件提供的平均CT值与标准CT值在不同大小、密度结节中均具有较大误差，不能应用于临床。

## Abstract (en)

**Background and objective:**

The computed tomography (CT) follow-up of indeterminate pulmonary nodules aiming to evaluate the change of the volume and CT value is the common strategy in clinic. The CT dose needs to considered on serious CT scans in addition to the measurement accuracy. The purpose of this study is to quantify the precision of pulmonary nodule volumetric measurement and CT value measurement with various tube currents and reconstruction algorithms in a phantom study with dual-energy CT.

**Methods:**

A chest phantom containing 9 artificial spherical solid nodules with known diameter (D=2.5 mm, 5 mm, 10 mm) and density (-100 HU, 60 HU and 100 HU) was scanned using a 64-row detector CT canner at 120 Kilovolt & various currents (10 mA, 20 mA, 50 mA, 80 mA, 100 mA, 150 mA and 350 mA). Raw data were reconstructed with filtered back projection and three levels of adaptive statistical iterative reconstruction algorithm (FBP, ASIR; 30%, 50% and 80%). Automatic volumetric measurements were performed using commercially available software. The relative volume error (RVE) and the absolute attenuation error (AAE) between the software measures and the referencestandard were calculated. Analyses of the variance were performed to evaluate the effect of reconstruction methods, different scan parameters, nodule size and attenuation on the RPE.

**Results:**

The software substantially overestimated the very small (D=2.5 mm) nodule's volume [mean RVE: (100.8%±28%)] and underestimated it attenuation [mean AAE: (-756±80) HU]. The mean RVEs of nodule with diameter as 5 mm and 10 mm were small [(-0.9%±1.1%) *vs* (0.9%±1.4%)], however, the mean AAEs [(-243±26) HU *vs* (-129±7) HU)] were large. The *ANOVA* analysis for repeated measurements showed that different tube current and reconstruction algorithm had no significant effect on the volumetric measurements for nodules with diameter of 5 mm and 10 mm (*F*=5.60, *P*=0.10 *vs* *F*=11.13, *P*=0.08), but significant effects on the measurement of CT value (*F*=34.79, *P* < 0.001 *vs* *F*=156.14, *P* < 0.001).

**Conclusion:**

An infinitesimally small errors of volumetric measurement of 5 mm or 10 mm nodule could achieved with very low current and ASIR reconstruction, suggesting a possibility of remarkable radiation dose reductions, while it is not applicable for 5 mm nodule. The attenuation acquired through three dimensional software has large measurement error and can not applied in clinical currently.

我国肺癌发病率及死亡率均居首位^[1]^。肺癌的早期诊断对提高生存率尤为重要^[2]^。肺结节的生长特性是判断其良恶性的重要指标之一，恶性结节常表现出相对持续快速增长的特性，而良性结节的生长速度则一般相对较慢^[3]^。目前临床上针对不定性小结节最常采用的策略是利用计算机断层扫描（computed tomography, CT）随访观察结节的容积变化。肺结节容积定量技术是近些年新研发的一种计算机后处理技术，其基本原理是利用肺结节与周围肺组织间的密度差，利用计算机软件将肺结节从肺实质中分割出来，自动重建出结节的三维立体图像并计算出基于体素的结节容积及CT值。对于非实性结节，在容积测量的基础上引入质量测量。质量的计算是基于结节的容积及CT值，具体方法是通过容积定量测出容积，将结节的CT值加上1, 000作为密度，二者的乘积转化为质量^[4]^。

随访研究的发展方向对肺结节的精确容积及CT值定量提出需求。同时，随访过程中的多次CT扫描必须要考虑到CT扫描的放射剂量，应尽量减小对受检者的放射损伤。基于上述原因，我们的研究旨在评估不同扫描条件对肺结节的容积及CT值测量的影响。

## 1 材料与方法

### 1.1 肺结节体模

肺结节模型是采用FUYO公司制造的体模模型（图 1）。该模型内固定四种直径（2.5 mm、5.0 mm、10.0 mm、20.0 mm）的球状物代表肺结节。每种直径的结节分别由亚克力、单体浇铸尼龙和聚丙烯三种不同材料制成，其CT值分别为-100 HU、60 HU、100 HU。另两个为直径10.0 mm的混杂密度结节，其平均CT值分别约为0 HU、-60 HU。模型内共有5种密度的16个结节，因为直径20 mm结节在临床中没有容积分析价值，另2种混杂密度结节不具有2.5 mm及5 mm直径的类型而无法对比，故仅纳入10.0 mm、5.0 mm、2.5 mm的不同密度结节。

**1:**
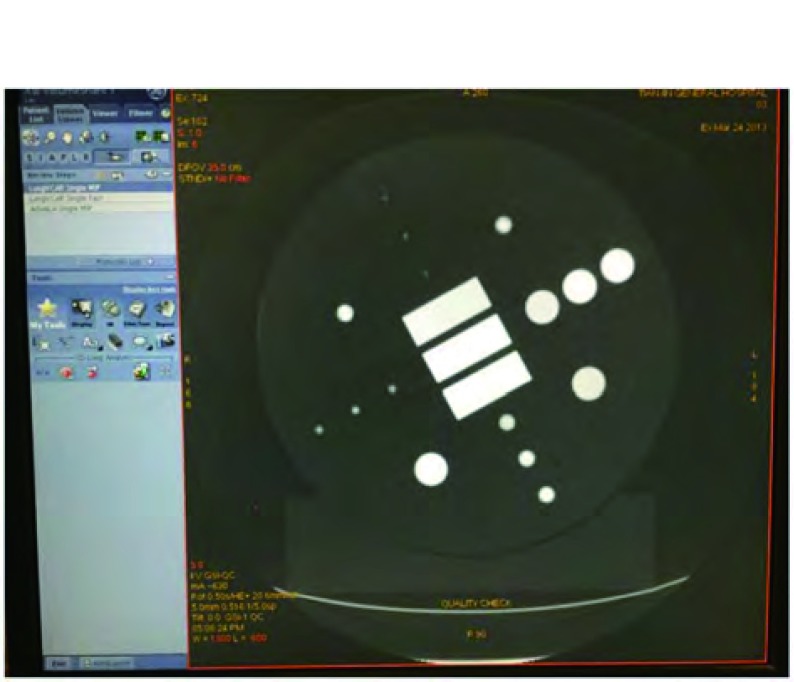
体模结节的CT扫描图 The CT image of the nodule phantom

### 1.2 检查方法

本研究使用64排螺旋CT机（GE Lightspeed VCT）对体模进行扫描，扫描范围自体模一端至另一端，螺旋扫描方式，120 kV，螺距1.375:1，机架旋转一周时间0.5 s，显示野（field of view, FOV）360 mm，图像矩阵512×512，默认重建层厚和重建间距均为1.25 mm，在管电流为10 mA、20 mA、50 mA、80 mA、100 mA、150 mA、350 mA分别进行扫描。扫描完成后，将数据利用传统滤波反投影法（filtered back projection, FBP）、自适应统计迭代重建技术（adaptive statistical iterative reconstruction, ASIR）（30%, 50%, 80%）分别进行4次重建，共获得28套CT图像。

### 1.3 容积分析及CT值测定

将CT图像传输到GE AW4.6工作站中，使用ALA软件对图像进行后处理及容积定量、CT值测量。选择要进行测量的一组轴位图像数据，找到体模结节的最大直径层面，选取待测结节，用鼠标单击结节的中心区，进入容积分析界面。软件自动实现对结节的分割并显示出结节的三维立体图像及其容积与平均CT值。在容积分析时，针对结节的大小及密度的不同，有三种模式可供选择，分别为磨玻璃密度结节模式、亚实性结节模式及实性结节模式。操作者在轴、矢及冠状位图像上观察结节分割情况，对分割不满意及未能显示的结节，可更换容积分析模式以达到最佳效果。最终对分割满意的结节，记录其容积V（单位为mm^3^）与平均CT值（HU）。每个结节可获得28组数据。

### 1.4 统计分析

所有统计分析都在统计学软件SPSS 17.0中完成，*P* < 0.05定义为差异具有统计学意义。肺体模结节的参考容积根据结节的直径按照球体的容积计算公式得出。计算容积的相对误差（relative volume error, RVE）的95%一致性区间^[5]^，RVE定义为测量值与参考值的差值与参考值的比值，计算CT值的绝对误差（absolute attenuation error, AAE）的95%一致性区间，AAE定义为软件测量的平均CT值与标准CT值的差值。以容积相对差值及CT值绝对差值作为自变量，管电流及重建算法作为组内影响因素，结节大小及密度作为组间影响因素，应用重复测量方差分析不同管电流及原始数据重建算法对节容积定量及CT值测量的影响。

## 2 结果

### 2.1 肺体模结节的分割与自动测量

28套CT图像中所有待测结节的测量均达到分割满意，其中直径为2.5 mm的小结节必须采用磨玻璃密度结节模式方可以实现容积分析，其余直径为5.0 mm及10.0 mm的小结节均使用实性结节模式。通过软件自动测量，分别记录每套CT图像中9个结节容积及平均CT值。

### 2.2 结节的容积测量相对误差及CT值测量绝对误差

根据9个结节在28套CT图像中所得的数据计算其容积RVE及CT值AAE（表 1）。直径为2.5 mm结节的容积测量相对误差（100.8%±28%）及CT值绝对误差（-756±80）HU均较大；直径为5 mm及10 mm结节的容积相对误差很小[(-0.9%±1.1%) *vs* (0.9%±1.4%)]，CT值绝对误差很大[(-243±26) HU *vs* (-129±7) HU]。其中直径为2.5 mm体模结节的容积被明显高估，且测量CT值与标准CT值相差悬殊。直径为5 mm及10 mm结节的测量容积几乎与标准容积相等，误差值很小，但测量CT值与标准CT值仍存在很大误差。

**1:** 结节的容积测量相对误差及CT值测量绝对误差 The relative error of volume and the absolute error of CT value of nodules

Standard diameter (volume)	Standard CT value	RVE			AAE	
		Mean (%)	SD (%)		Mean (%)	SD (%)
2.5 mm (8.17 mm^3^)	100 HU	105.5	17.7		-838.4	7.4
	60 HU	121.2	13.3		-760.0	8.3
	-100 HU	75.7	10.1		-650.2	7.7
5.0 mm (65.4 mm^3^)	100 HU	-0.3	1.1		-272.1	9.1
	60 HU	0.4	1.2		-246.3	7.8
	-100 HU	-2.7	1.5		-211.3	4.6
10.0 mm (523 mm^3^)	100 HU	0.3	0.3		-132.6	4.4
	60 HU	-0.6	0.3		-135.6	5.0
	-100 HU	-2.3	0.3		-122.0	4.5
CT: computed tomography; RVE: relative volume error; AAE: absolute attenuation error.						

将所得原始数据按照不同的管电流及重建算法进行分类处理（图 2-图 5），结果显示：对于直径为2.5 mm的结节在7种不同管电流及4种不同重建算法下，结节的容积相对误差及CT值绝对误差均较大。对于直径为5.0 mm及10.0 mm的结节，不同管电流及重建算法对结节的容积相对误差几乎没有影响；但CT值绝对误差较大。

**2:**
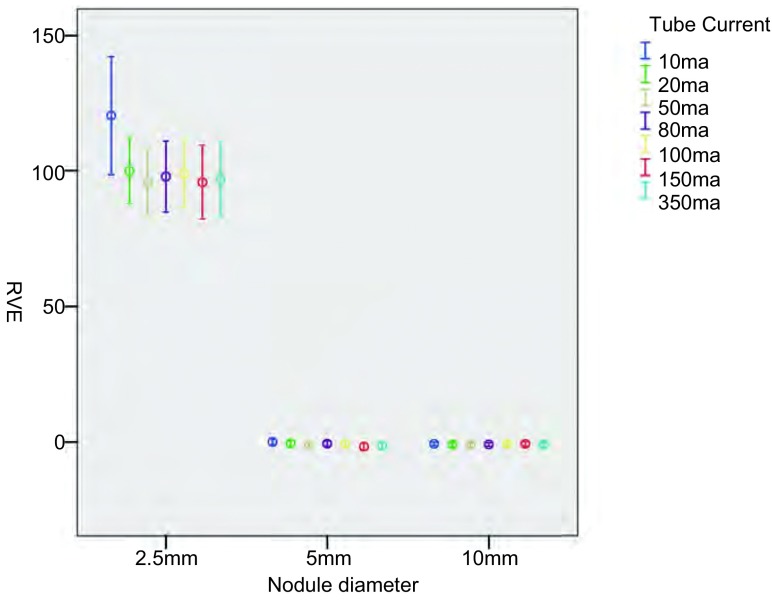
不同管电流对结节容积相对误差 The RVE for different cube current

**3:**
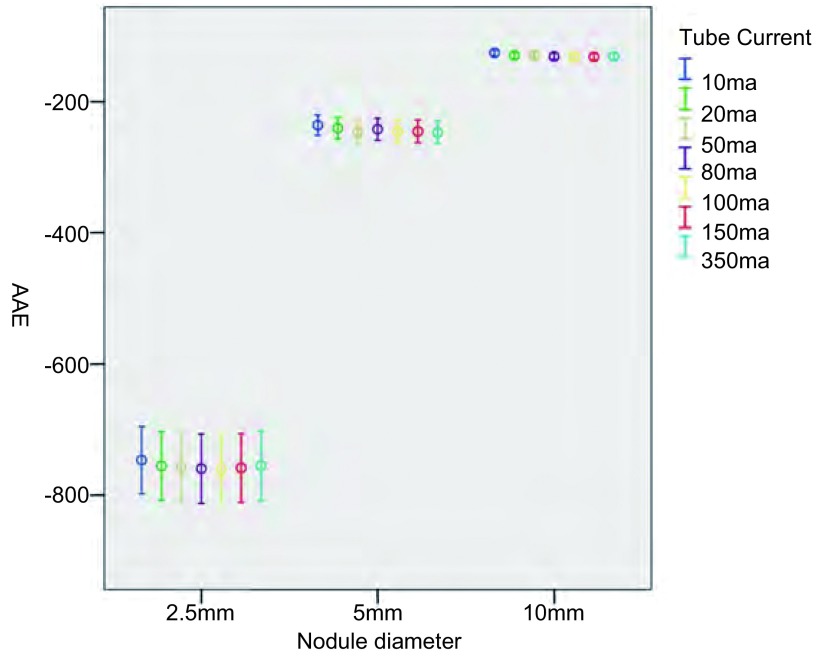
不同管电流对结节CT值绝对误差 The AAE for different cube current

**4:**
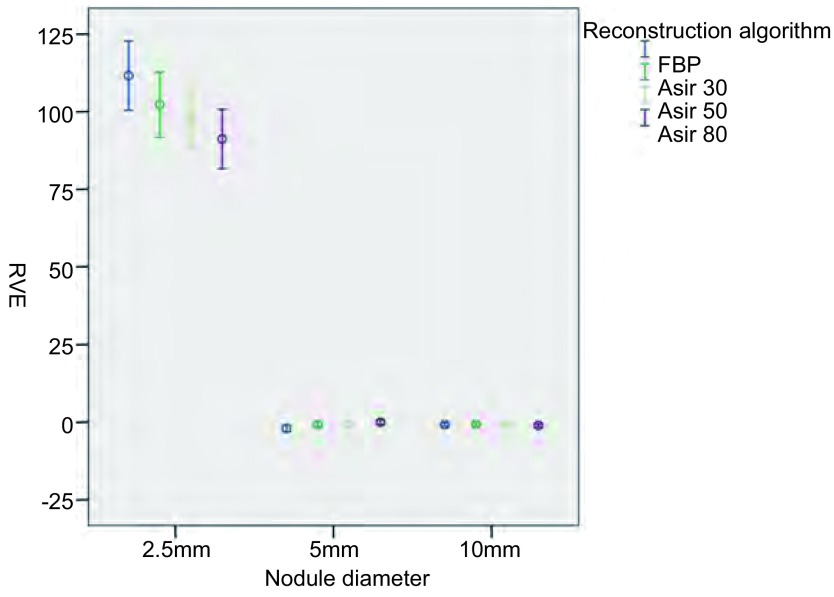
不同重建算法对结节容积相对误差 The RVE for different reconstruction algorithm

**5:**
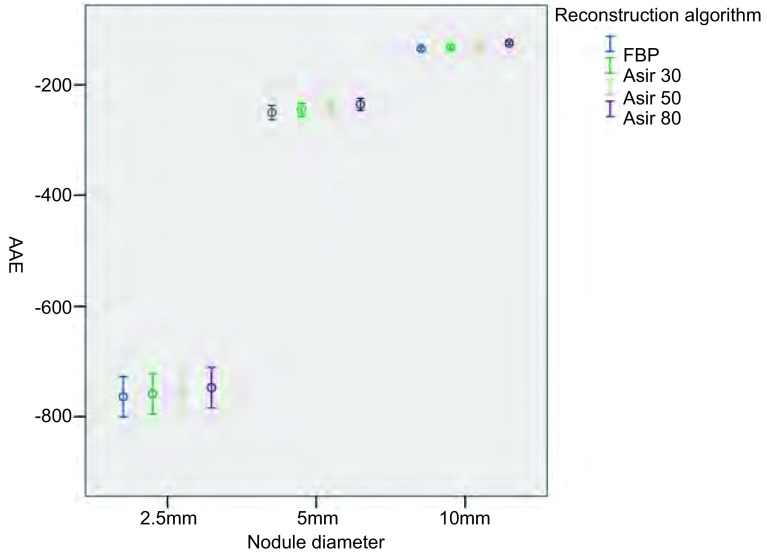
不同重建算法对结节CT值绝对误差 The AEE for different reconstruction algorithm

### 2.3 结节容积测量相对误差及CT值测量绝对误差的影响因素

由于直径2.5 mm的结节在7种不同管电流及4种不同重建算法下，结节的容积相对误差及CT值绝对误差均较5 mm及10 mm结节明显增大，将其纳入重复测量方差分析意义不大，故仅对直径为5.0 mm及10.0 mm的结节做进一步方差分析，结果显示：不同管电流及原始数据重建算法对容积相对误差没有显著影响（*F*=5.6, *P*=0.10; *F*=11.13, *P*=0.08），对CT值绝对误差有显著影响（*F*=34.79, *P* < 0.001; *F*=156.14, *P* < 0.001）。

## 3 讨论

我们的研究发现直径为2.5 mm的结节，无论采用何种扫描条件，软件的容积分析均存在很大误差。对于5 mm及10 mm的结节，不同管电流及重建算法对结节的容积定量没有影响。软件测量所得的CT值在所有结节中均存在较大误差。

### 3.1 结节的容积测量影响因素

当结节直径为2.5 mm时，容积测量误差很大，且往往被高估，其主要原因在于容积自动分析时的部分容积效应。部分容积效应是指CT图像上各个像素的数值代表相应单位组织全体的平均CT值，它不能如实反映该单位内各种组织本身的CT值。在CT图像中结节边缘所经过的每一个像素的CT值是结节的真正部分和周围结构的平均值，如果平均值超过软件采用的阈值，则被纳入容积分割的范围，反之则被除外。我们的研究发现直径2.5 mm的结节容积被明显高估。其原因在于相对于重建视野（FOV）360 mm、矩阵512×512，CT图像的像素大小约为0.7 mm×0.7 mm。直径2.5 mm的结节其最大层面在X、Y轴可完全或部分占据4个-5个像素，其中2个-3个像素为受部分容积效应影响的像素。此外，软件容积分析对这种小结节仅能采用针对磨玻璃密度结节的算法，其容积分割的阈值较低。因此，结节部分占据的像素可能均被纳入容积分割中，导致测量容积增大。相对较大的结节，一方面其占据的像素较多，受部分容积影响的像素在总体中所占比例较小，另一方面，较大结节容积分析采用的是针对实性结节的算法，其阈值较高，被结节部分占据的像素会部分纳入分割，部分被排除。因此，结节的容积分析误差在较大结节中很小，而在较小结节中很大。

### 3.2 结节CT值测量的影响因素

三维软件自动分析结节的CT值和真实CT值相差较大，一方面这可能与软件结节分割所采用的阈值法有关，软件分割的阈值在不同算法中具有差异，一般来说均在-400 HU以下。因此，结节分割常会包含部分CT值较低的像素，造成测量的误差。此外，两种直径相同但不同密度的体模结节的测量值的差值与真实值的差值（表 1）也不一致，我们估计可能与软件的算法有关。目前的软件尚无法提供可靠的三维整体CT值，故无法应用于临床。我们建议采用传统的ROI测量法。因此，三维软件如要更精确的进行CT值测量，则需要对像素识别及算法做出调整。

### 3.3 低剂量CT扫描和使用迭代重建算法的可行性

无论在肺癌筛查还是结节随访中，容积定量都是相当关键的，同时容积倍增时间也是辅助判断结节良恶性的重要指标^[6]^。本实验结果显示，对于直径为5.0 mm及10.0 mm的结节，不同管电流及重建算法对结节的容积定量没有影响。那么就为在肺癌筛查及结节随访中选择最合适的管电流及重建算法提供了可行性。

根据美国NCCN指南建议，推荐低剂量CT作为肺癌的主要筛查手段^[7]^。根据我们的实验结果，直径5 mm以上结节，降低管电流对容积定量影响很小。CT扫描的剂量与电流呈正相关，因此，在不影响结节容积定量的前提下，降低电流可显著降低辐射剂量，减少受检者的放射损伤，对肺癌的预防有着极为重要的意义。尽管我们的研究提示10毫安的管电流对结节的容积分析没有显著影响，但其受结节体模整体密度较低的影响，人体肺组织外尚包含骨骼及肌肉组织，会造成X射线的衰减，因此在临床应用中尚需要适当提高管电流。

实验中使用了4种重建算法，包括传统滤波反投影法（FBP）及自适应统计迭代重建技术（ASIR）。ASIR技术自2009年底用于临床，其特点在于噪声消除、伪影抑制以及双能成像。国外很多研究表明，采用该技术可以大大降低受检者接受的辐射剂量，而扫描中所获得图像质量及影像信息完全可以满足诊断要求^[8-10]^。ASIR与FBP相比最大的优势是可以降低由于辐射剂量的降低而增加的噪声，这就可以弥补使用低剂量CT扫描时，降低管电流等而增加的噪声。同时由于不同重建算法对结节容积定量影响很小，故可以使用自适应统计迭代重建技术配合低电流CT扫描，最终达到降低辐射剂量的目的。

综上所述，使用较低的电流及自适应迭代重建算法实现低剂量扫描对直径为5.0mm及10.0 mm的结节容积定量没有影响，对2.5 mm结节的容积定量误差较大，测量的容积明显被高估，不建议对此类结节进行容积分析。三维软件自动分析结节的CT值和真实CT值相差较大，所以目前临床上还是要采用传统的手动测量法。
